# Frequency of rare and multi viral high-risk HPV types infection in cervical high grade squamous intraepithelial lesions in a non-native dominant middle eastern country: a polymerase chain reaction-based pilot study

**DOI:** 10.1186/s13000-018-0716-x

**Published:** 2018-06-26

**Authors:** Alia Albawardi, M. Ruhul Quddus, Shamsa Al Awar, Saeeda Almarzooqi

**Affiliations:** 1 Pathology Department, College of Medicine and Health Sciences, UAE University, Al Ain, United Arab Emirates; 2 Department of Pathology, Women & Infants Hospital/Alpert Medical of Brown University, Providence, RI 02905 USA; 3 Obstetrics and Gynecology Department, College of Medicine and Health Sciences, UAE University, Al Ain, United Arab Emirates

**Keywords:** Cervical cancer, HPV, Papillomaviridae, UAE

## Abstract

**Background:**

The incidence of abnormal cervical smears in the United Arab Emirates (UAE) is 3.6%. Data regarding specific high-risk HPV (hrHPV) genotypes are insufficient. Identification of hrHPV subtypes is essential to allow formulating effective vaccination strategies.

**Methods:**

A total of 75 archival cervical cone biopsies with HSIL or higher lesions (2012–2016) were retrieved from a tertiary hospital, including HSIL (*n* = 70), adenocarcinoma in-situ (*n* = 1) and squamous cell carcinoma (*n* = 4). Five tissue sections (10-μ-thick each) were cut and DNA extracted using the QIAamp DNA FFPE Tissue Kit. GenomeMeTM’s GeneNavTM HPV One qPCR Kit was used for specific detection of HPV 16 and 18; and non-16/18 samples were typed by GenomeMeTM’s GeneNavTM HPV Genotyping qPCR Kit.

**Results:**

Median age was 34 years (range 19–58) with 70% UAE Nationals. hrHPV detected were 16, 18, 31, 33, 35, 39, 45, 51, 52, 58, 59, 66 & 68. hrHPV testing was negative in 12% of cases. Most common types were HPV 16 (49%), HPV 31 (20%) and HPV 18 (6.6%). hrHPV 16 and/or 18 represented 56% and rare subtypes 32%. Co-infection was present in 16%. Eight cases had two-viral subtype infections and 4 cases had 3 subtype infections. Multi-viral HPV infection was limited to hrHPV 16, 18, 31 & 33 subtypes.

**Conclusions:**

Infection by non HPV 16/18 is fairly common. A higher than expected incidence of rare subtype (20% hrHPV31) and multi-viral hrHPV (16%) were detected. This finding stresses the importance of this pilot study as currently only quadravalent vaccine is offered to control the HPV infection in the UAE population.

## Background

Cervical cancer is the third most common cancer in females in the United Arab Emirates as per 2014 cancer incidence report in the emirate of Abu Dhabi [1]. It is the fourth leading cause of cancer-related mortality in the UAE [2]. Human Papillomavirus (HPV) infection has been established as an etiologic agent in the pathogenesis of cervical neoplasia [3]. HPV infection is reported in 80% of the cases with low-grade squamous intraepithelial lesions (LSILs) and 90% of cases with high-grade squamous intraepithelial lesions (HSILs) [4].

The reported incidence of cervical abnormalities in cervical smears among women in the UAE is 3.6% in one study [5]. The author reported lesions including LSIL, HSIL, ASCUS (atypical squamous cells of undetermined significance) and glandular abnormalities. However, tissue biopsy confirmation of the findings was not reported by the investigators and characterization of HPV subtypes was not performed - [5]. A comparable overall incidence of 3.3% was also reported in a retrospective study performed on cervical Pap smears on 602 UAE and immigrant women in 2012. In this study ASCUS represented 1.8%, LSIL 1.2%, and HSIL 0.3%. The ASCUS:SIL ratio was 1.2 [6]. The ASCUS:SIL ratio at our lab was 1.9 in 2017 (non-published data).

Infrequent reports on studies identifying hrHPV types are available from the countries with somewhat similar geography and demography to the UAE. One such report included women presenting to an obstetrics and gynecology department at a hospital in Bahrain [7]. It reported the following epithelial abnormalities on Pap smears from 1082 women: LSIL 1.94%, HSIL 0.46%, ASCUS 0.18% and invasive lesions in 0.64%. The most common hrHPV type was HPV 16 and 52. In addition, HPV 18, 31, 33, 51, 44, 45, 56 and 59 were detected in their study population. The sample included both Bahraini women (32%) and other nationalities [7]. Hajja et al. has demonstrated the presence of hrHPV genotypes 16,18,45,62 and 53 in a smaller sample of 100 women attending a gynecology clinic in Bahrain [8]. In Saudi Arabia, the prevalence of HPV infection was 9.8% among Saudi women seen during routine screening [9]. The most common hrHPV were 68/73 (12%), HPV 18 (9.8%) and HPV 16 (7.3%) [9]. In a population based study performed on 799 Iranian women, cervical abnormalities were detected in about 5% of Pap smears. hrHPV genotypes were found to be HPV 16, 18, 31, 33, 51, 56 and 66 [10]. A molecular study conducted in Pakistan on a sample of normal cervical smears demonstrated the following hrHPV types in their sample: HPV 33 (8.33%), HPV 18 (6.25%) and HPV 16 (4.17%) [11].

In the ATHENA trial conducted in the USA on a large screening population, HPV 16 was reported as the most commonly associated hrHPV genotype with high-grade cervical lesions. In addition, the report also found an association of hrHPV 18 with half of the cases of in-situ and invasive adenocarcinoma of the uterine cervix [12].

Data regarding the exact prevalence of various hrHPV types in the UAE population are limited. In 2008, the Health Authority of Abu Dhabi (HAAD) introduced HPV vaccination to all female students between the ages of 15–17. In addition, regular screening is recommended every 3–5 years to women aged 25–65 [13]. Both FDA approved vaccines, e.g., quadrivalent (Gardasil, Merck) and bivalent (Cervarix, GlaxoSmithKline Biologicals) are approved and administered in the UAE. Gardasil vaccine targets HPV 6, 11, 16, and 18 with an aim to reduce the incidence of genital condylomas caused by HPV 6, 11 and cervical/vaginal/vulvar intraepithelial neoplasias (CINs, VAINs, VIN) and carcinomas caused by the most frequently implicated subtypes of hrHPV 16, 18. Cervarix is administered in the UAE for the prevention of cervical carcinoma that targets HPV 16 and 18 [14, 15].

Multiplex Real-Time Polymerase Chain Reaction-based (PCR) technique has recently been used to genotype hrHPV in the UAE population which allows simultaneous detection of multiple hrHPV genotypes [16].

Knowledge of the specific hrHPV subtypes in the UAE population would not only help guide the formulation of proper cancer screening recommendations, but also assist in directing vaccination program strategies should the infection patterns deviate from the known national/regional/ and international epidemiologic data.

## Methods

### Subjects

Women diagnosed with cervical squamous intraepithelial neoplasias and cervical carcinomas were retrieved from the archives of the Department of Pathology at a tertiary care hospital (Tawam Hospital, Abu Dhabi) during the period from 2012 to 2016. Hematoxylin and eosin stained slides were reviewed by two of the investigators (MRQ and SAA) to confirm the diagnosis. Paraffin tissue blocks were retrieved on all cases except those with insufficient residual tissue. Cases were classified based on morphologic criteria for cervical neoplasia according to the World Health Organization (WHO) Classification of Tumors of Uterine Cervix [4]. Demographic and pathological data including age, nationality, pathological diagnosis and tumor-related histological features where applicable (histological subtype, tumor size, grade, stage) were all documented.

### DNA extraction and HPV typing

Five representative tissue sections (each 10-μ-thick) were obtained from archival formalin fixed paraffin embedded (FFPE) tissue and placed in Eppendorf tubes. Sterile precautions were taken to avoid cross-contamination including changing cutting blades between cases and changing hand gloves. DNA was extracted using the QIAamp DNA FFPE Tissue Kit (QIAGEN Inc., Valencia, CA 91355, Cat No. 56404) at Women & Infants Hospital, Providence, RI, USA following the protocol recommended by the manufacturer. hrHPV genotyping was initially performed using multiplex PCR followed by Tag/Capsure probe hybridization at Memorial Hospital of Rhode Island, USA, a laboratory facility of Women & Infants Hospital. Subsequently the findings were ratified using a second detection method, GenomeMeTM’s GeneNavTM HPV One qPCR Kit (GenomeMe, Richmond, BC, Canada) which specifically detected HPV 16 and18 and non-specially detected other rare subtypes. Subsequently all non-16/18 positive samples were typed on-site at GenomeMe, Canada, Richmond, BC, Canada using their GeneNavTM HPV Genotyping qPCR Kit.

## Results

**Fig. 1:**
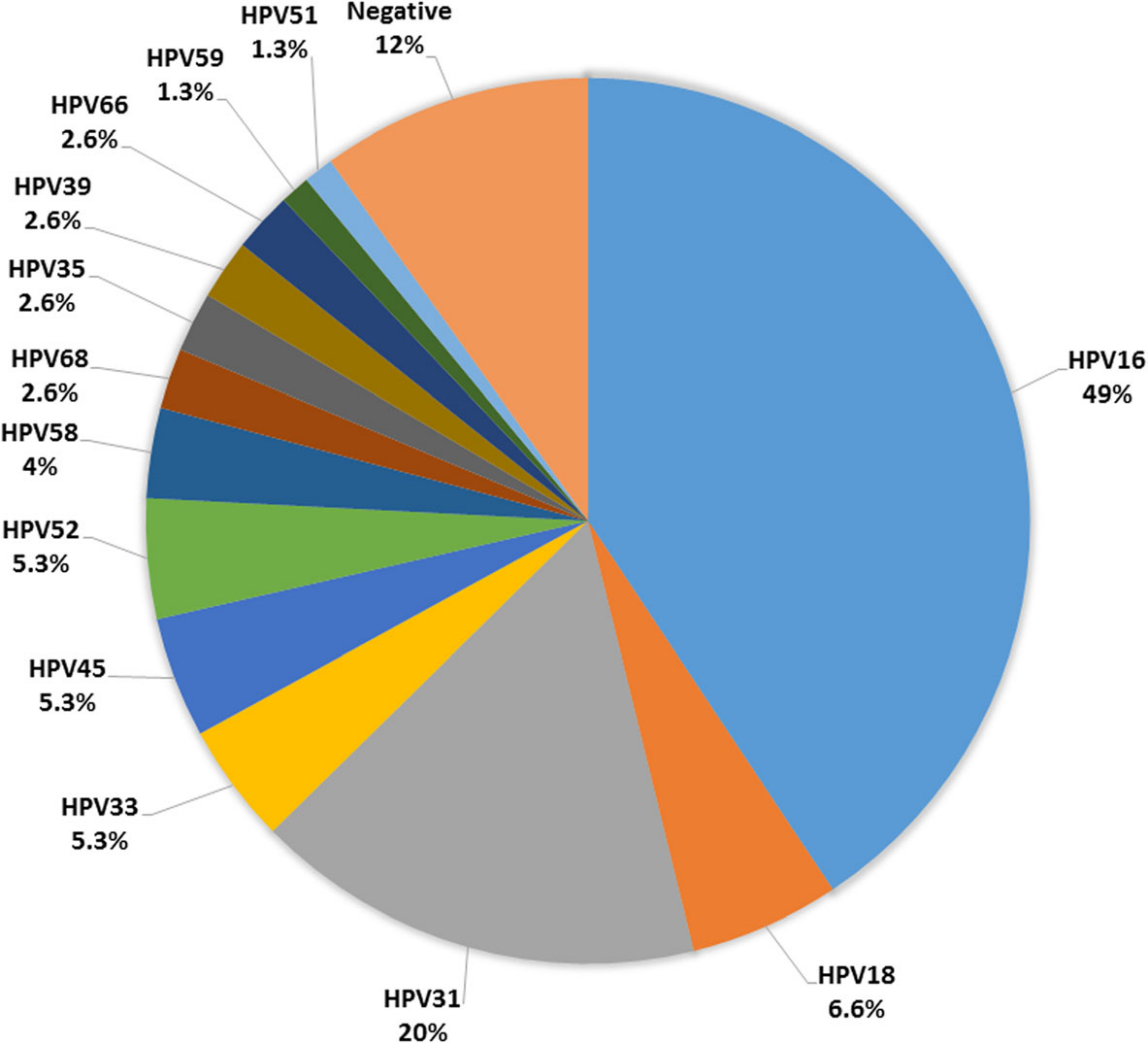
Frequency of HPV infection among 75 cases

The study was based on a total of 75 cases including HSIL (*n* = 70), adenocarcinoma in-situ (*n* = 1) and invasive squamous cell carcinoma (*n* = 4). The median age for HSIL and higher lesions was 34 years (range 19–58 years). UAE nationals constituted 53 cases (70%) of the study sample. Overall, hrHPV types detected were 16, 18, 31, 33, 35, 39, 45, 51, 52, 58, 59, 66 and 68 (Table 1 and Fig. 1). Nine of 75 (12%) HSIL cases were negative for hrHPV. Most common types were HPV 16 (49%), HPV 31 (20%) and HPV 18 (6.6%). HPV 16 and 18 were identified in 56% and other types in 32%. Table 2 illustrates HPV distribution among different age groups. Patients between 25 and 45 years had a higher frequency of HPV 16 and 31. HPV 16 was present across all age groups. Co-infection was seen in 12 of the 75 cases (16%) (Table 3). Eight cases had two viral subtype infections and four cases had three subtype infections. Multi-viral HVP infection was limited to cases with HPV 16 (*n* = 5), 18 (*n* = 2), 33 (*n* = 1) & 31 (*n* = 4) only. Co-infection consisted of genotypes of the same species in four cases. The remaining cases were infected by a combination of HPV genotypes consisting of different species (67% of cases). The species detected in this study sample included α9 species (HPV 16, 31, 33, 52), α7 species (HPV 18, 39, 59, 68), α6 species (HPV 66) and α5 species (HPV 51).

**Table 1:** Frequency of hrHPV types in 75 cases of HSIL/AIS/Invasive carcinoma

Serotype detected	*n*^a^ (%)
HPV16	37 (49%)
HPV18	5 (6.6%)
HPV31	15 (20%)
HPV33	4 (5.3%)
HPV45	4 (5.3%)
HPV52	4 (5.3%)
HPV58	3 (4%)
HPV68	2 (2.6%)
HPV35	2 (2.6%)
HPV39	2 (2.6%)
HPV66	2 (2.6%)
HPV59	1 (1.3%)
HPV51	1 (1.3%)
Negative	9 (12%)
Two serotypes	8 (10.6%)
Three serotypes	4 (5.3%)

^a^*n* number of cases

**Table 2:** HPV genotypes by age group

Age group	HPV genotypes
< 25	31
	16
25–34	58
	45
	33
	35 (*n* = 2)
	18 (*n* = 2)
	31 (*n* = 8)
	16 (*n* = 19)
35–44	18
	58 (*n* = 2)
	45(*n* = 2)
	31 (*n* = 4)
	16(*n* = 14)
45–54	45
	33
	18 (*n* = 2)
	16(*n* = 2)
+ 55	16

**Table 3:**
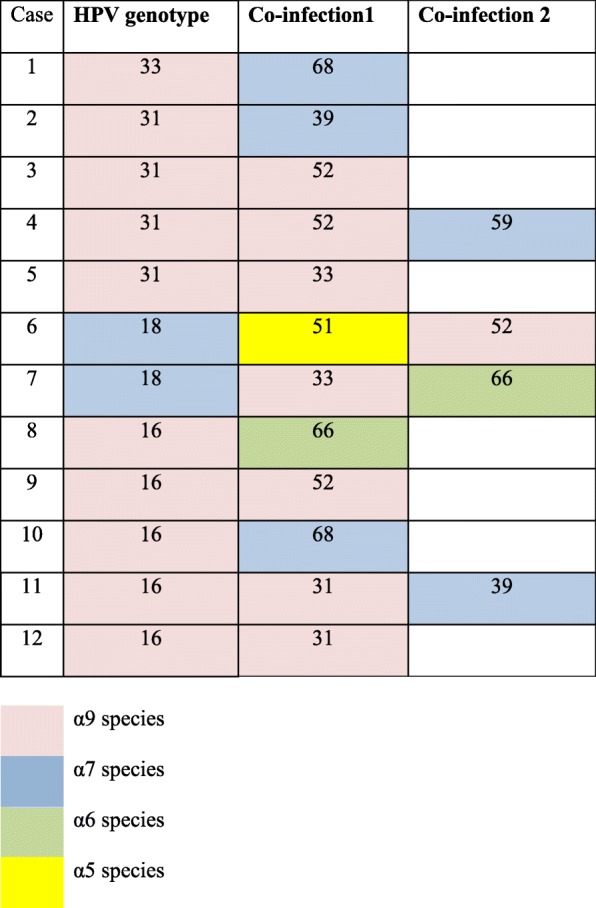
HPV genotypes in cases of co-infection highlighing different HPV species in the 12 cases with co-infection

The results of this study were presented in part at the 106^th^ Annual Meeting of the USCAP in March 2017, San Antonio, Texas, USA [17].

## Discussion

High-risk Human Papilloma Virus infection, the single most important etiologic agent for uterine cervical carcinomas, appears to show regional variation in the prevalence of its subtypes. Worldwide, the most common HPV genotypes are HPV 16/18 detected in up to 70% of cervical cancers [18]. The assumption that HPV 16 and 18 are also the most common genotypes in the UAE and other regions may not be accurate. Epidemiologic variability of infection pattern is more likely [19]. This is evident from various publications in the region and worldwide [9, 11].

The following reports published from Asia, South America, Europe, Africa, and North America also ratify this view. In Turkey, HPV 16 (20.7%) represented the most common HPV genotype in a screening sample of one million women. It was followed by HPV 51 (10.8%), 31 (8.7%), 52 (7.1%) and 18 (5.1%) [20]. Similarly, a Malaysian study found HPV 16, 52 and 58 to be the most common types in their study sample of 1293 women [21].

A study in Bahrain reported HPV 16 and 52 as the most common types [7]. In a similar study on 298 women in Kuwait, the authors screened ThinPrep smears during routine gynecological examinations and genotyped HPV using PCR-based technique [22]. The study found that HPV 16 was the most common HPV type (24.3%). Other frequent types included HPV 11 (13.8%), HPV 66 (11.2%), HPV 33 (9.9%), HPV 53 (9.2%), HPV 81 (9.2%), HPV 56 (7.9%) and HPV 18 (6.6%) [22]. In Saudi Arabia, a cross-sectional study of 417 women (mean age 41.9) attending routine gynecological evaluations were screened for hrHPV [9], revealing HPV DNA in 9.8% (41 cases) of the study sample. The majority of their cases (77%) were Saudi women. The most common hrHPV types were hrHPV68/73 (12.2%) followed by HPV 18 (9.8%) and HPV 16 (7.3%). There were only 41 positive cases for HPV from the 417 cases assessed. Other HPV types detected were HPV 31 (4.9%), HPV 51 (4.9%), HPV 52 (4.9%), HPV 39 (2.4%), HPV 56 (2.4%) and HPV 58 (2.4%). The low-risk HPV types included HPV 6, 42, 53, 54, 11, 40, 70 and 74 in order of frequency [9]. The study, although offering a preview of the incidence of HPV in the Saudi population, may not hold true when a larger population based study is undertaken [9]. An Iranian study reported HPV 16 and 18 as the most common genotypes in their study population [10].

A study in Xinjiang (in north-western China) performed using routine Pap smear found that HPV 16, 58 and 39 are the most common genotypes [23]. In contrast, HPV 16 and 18 were the most common genotypes detected in a sample of 2309 cervical cancer patients in western China, followed by HPV 58, 53, and 33 [24]. Thus, even within the same country, there might be regional variations.

HPV 16 (38.9%) and HPV 58 (19.5%) were found to be the most common HPV genotype in cervical lesions and cervical cancer in the coastal region of Ecuador [25]. In the US, HPV 16 is the most common genotype in a screening population reported by the ATHENA trial [26]. In North America, HPV 16 constituted 5.8% and HPV 18 2.1% followed by HPV 52 2.0%. In contrast, in continental Europe HPV 16 constituted 4.8% and HPV 31 2.3% followed by HPV 18 0.9%. In African continent, HPV 16 (3.5%) and HPV 52 (2.4%) were the most prevalent types [27].

A recent study in the UAE on cases of ASCUS (atypical squamous cells of undetermined significance) demonstrated the presence of hrHPV in 17.9% of cases [28]. The authors also reported hrHPV 16 as the most common type isolated in ASCUS cases for patients aged 25–34 years. Interestingly the authors found that hrHPV 18 was more frequent in patients above 64 years. In addition, other hrHPV types were more frequent in their cases of ASCUS in patients between 25 and 45 years. The exact frequencies and genotypes, however, were not reported. The findings are in keeping with the current report showing hrHPV 18 as a less frequent hrHPV type in the UAE population [28]. Similarly, Krishnan and Thomas reported HPV 16 to be the most common HPV type in cases with abnormal Pap smear [29]. Likewise, hrHPV 18 was not the second most frequent type. The following hrHPV types (51, 31, 66, 56 and 59) were reported by all these authors [29].

Despite many publications on HPV genotypes in different regions, there are still some gaps in available data on different HPV genotypes in various geographic regions and even within different regions of the same country. This variability may reflect differences in geographic, ethnic, socio-cultural, and religious practices of the region. In a recent study in the USA found that HPV 16 was less prevalent in Hispanic and Black women compared to White women [26].

In the current study, HPV 16 was detected in the majority of cases (49%), whereas HPV 18 was detected only in 6.6% of the study sample, a significant deviation from what has been reported from the western world. Bruni et al. performed a meta-analysis on HPV infection prevalence dividing the studies into five geographic regions: worldwide, Americas, Africa, Asia and Europe. It included studies addressing both low and high risk HPV. They found that the most common HPV types worldwide were HPV 16 (3.2%) and HPV 18(1.4%) [27]. In northern America, HPV 16 constituted 5.8% and HPV 18 2.1% followed by HPV 52 2.0%. In contrast, in continental Europe HPV 16 constituted 4.8% and HPV 31 2.3% followed by HPV 18 0.9%. In African continent, HPV 16 3.5% and HPV52 2.4% were the most prevalent types [27].

The detection of HPV 31 in 20% of the samples examined in this study has potential implications in terms of vaccination strategies currently practiced here in the UAE. Currently, the two approved and available vaccines are the bivalent and the quadravalent vaccines which do not offer direct protection against HPV 31 [13–15]. Epidemiological studies are showing a trend of declining incidence of SIL and AIS in vaccinated subjects, and in addition, offering possible partial protection against some rare HPV types because of cross antigenicity [30]. Gardasil9, targets Types 6, 11, 16, 18, 31, 33, 45, 52, and 58 [31]. Thus, it appears reasonable to speculate that Gardasil9 might be a better alternative for the local population as HPV 31, detected in 20% of current study, is not one of the HPV subtypes to be directly protected by the two vaccines administered in the UAE currently.

Co-infection represented 16% of cases in the current study. It included cases infected with HPV 16, 18, 31, and 33. Infection with multiple species was present in 67% of the cases with co-infection. A study from India detected multiple genotypes in 23.41% of their HPV positive cases [32]. Interestingly, infection with multiple genotypes of α9 and α7species excluding HPV 16 and HPV 18 were associated with an increased risk of cervical cancer with an odds ratio of 5.3 and 2.5 respectively [32]. This finding is pertinent in formulating an effective vaccination strategy of any country or region. Quddus et al. detected co-infection in 32% of cases with adenosquamous carcinoma of the cervix in USA [33]. Hui et al. recently reported prevalence of multiviral HPV infection involving multiple anatomic sites of the lower female genital tract of the same individual and as high as 5 different subtypes of hrHPV were reported to be identified in an individual case in that series [34].

Ginindza et al. found that infection with multiple hrHPV increases in patients with decreased immunity. In their study including HIV patients the prevalence of multiple hrHPV infection was 27.7% compared to 12.7% in non-HIV patients [35]. It has been postulated that co-infection may propel carcinogenesis based on the association of multiple HPV genotype infection with cervical carcinoma [35].

All these studies reveal considerable geographic variation within the region and also from the Western studies. Other studies in the region demonstrated different results. It also appears that the rare subtypes appear to be prevalent in this region with different cultural and religious backgrounds. As each country in this region, or for that matter any other region of the world, is governed by independent administration despite their proximity it is imperative that the exact prevalence of hrHPV in a particular country/region should be available for effective control of this disease.

The current pilot study is based on a limited number of samples. The finding ought to be ratified by larger, multicenter studies to get an actual overall incidence of the HPV subtypes in the country. The current study addressed cases that presented with HSIL or higher lesions. Thus, data on HPV type prevalence might be skewed toward the more aggressive lesions. One would argue that from a clinical perspective, the more aggressive lesions are those that need intervention to prevent progression and reduce the incidence of cervical cancer related mortality and morbidity. The sample of HSIL positivity might indicate that those cases with the less aggressive HPV types did not progress and may have resolved without progression. Thus, those cases would not need attention when vaccination and screening program recommendations are being implemented in the country.

The incidence of hrHPV among the UAE population emphasizes the need for qualified well-trained healthcare professionals and gynecologists, who can perform colposcopic examination, detect and manage the disease appropriately; unfortunately, that need that has not been met here as off yet as reported by Ghazal-Aswad et al. [36].

It is noteworthy to mention that even after using the most sensitive techniques available now, about 12% of UAE women studied did not show any detectable hrHPV in the tissues samples examined. This may imply that other carcinogenic factors may be responsible for these or there are more yet unrecognized hrHPV.

The current findings are significant for the UAE population where the quadrivalent vaccine is currently used. This entails the possibility of limited protection in at least 20% of cases infected with HPV 31. In addition, the presence of co-infection by different HPV species warrants recommendation of vaccines with a wider coverage. Results will impact future screening and prevention recommendation by the Cervical Cancer Prevention Taskforce. This study will serve as a foundation for a larger study which appears prudent to ratify our findings.

It is worth stating that risk of an HSIL lesion developing into invasive cancer depends to a large extent on the type of HPV. It has been shown that HPV 16 and 18 are detected in higher percentages in cases of invasive cervical squamous cell carcinoma in comparison to cases of LSIL. In addition, HPV 33 and 45 are similarly detected in invasive lesions. HPV 31 has an odds ratio of 3.4 of developing invasive cervical cancer. Thus, HPV type virulence is another consideration when vaccination and screening guidelines are addressed [3].

## Conclusion

In conclusion, this pilot study illustrates that HPV 16 and 31 are the most frequent HPV genotypes in cervical neoplasia in the UAE population. Co-infection with multiple genotypes is present in 16% of cases and consists of different HPV subtypes. Should these findings hold true on a larger population-based study it would be imperative to introduce a vaccine which would offer direct protection against both common and some of the rare subtypes of HPV infection in the United Arab Emirates.

## Abbreviations

ASCUS: Atypical squamous cells of undetermined significance
CIN: Cervical intraepithelial neoplasias
FFPE: Formalin fixed paraffin embedded
HAAD: Health Authority of Abu Dhabi
HPV: Human Papillomavirus
hrHPV: High-risk HPV
HSILs: High-grade squamous intraepithelial lesions
LSILs: Low- grade squamous intraepithelial lesions
PCR: Polymerase Chain Reaction-based
UAE: United Arab Emirates
VAIN: Vaginal intraepithelial neoplasias
VIN: Vulvar intraepithelial neoplasias
WHO: World Health Organization
